# *CIC*-Rearranged Sarcoma: A Clinical and Pathological Study of a Peculiar Entity

**DOI:** 10.3390/diagnostics15141758

**Published:** 2025-07-11

**Authors:** Ward Maaita, Nabil Hasasna, Sameer Yaser, Yacob Saleh, Ramiz Abu-Hijlih, Wafa Asha, Hadeel Halalsheh, Samer Abdel Al, Maysa Al-Hussaini, Omar Jaber

**Affiliations:** 1 Department of Pathology and Laboratory Medicine, King Hussein Cancer Center, Amman P.O. Box 1269, Jordan; wardmaaita@icloud.com; 2 Department of Cell Therapy and Applied Genomics, King Hussein Cancer Center, Amman P.O. Box 1269, Jordan; nh.12632@khcc.jo (N.H.); mhussaini@khcc.jo (M.A.-H.); 3 Department of Medical Oncology, King Hussein Cancer Center, Amman P.O. Box 1269, Jordan; syaser@khcc.jo (S.Y.); ds.10117@khcc.jo (Y.S.); 4 Department of Radiation Oncology, King Hussein Cancer Center, Amman P.O. Box 1269, Jordan; rhijlih@khcc.jo (R.A.-H.); wa.11007@khcc.jo (W.A.); 5 Department of Pediatrics, King Hussein Cancer Center, Amman P.O. Box 1269, Jordan; hadeelhalalsheh@khcc.jo; 6 Department of Pediatrics, The University of Jordan, Amman P.O. Box 1269, Jordan; 7 Department of Surgery, King Hussein Cancer Center, Amman P.O. Box 1269, Jordan; da.10670@khcc.jo

**Keywords:** sarcoma, *CIC*, *DUX4*, translocation, undifferentiated small round cell

## Abstract

**Background**: *CIC*-rearranged sarcoma is a rare and aggressive type of undifferentiated round cell tumor characterized by *CIC* gene fusion, most commonly *CIC::DUX4.* This study presents a series of eleven cases, highlighting their clinicopathological features. **Methods**: Pathology records (2019 to 2024) were searched using “sarcoma with *CIC*”, identifying eleven cases, of which seven referred cases were initially misdiagnosed. Pathological and clinical analysis was conducted. Treatment was dictated upon multidisciplinary panel discussion based on tumor stage. Follow-up data (1–25 months) was available for all patients. **Results**: The cohort included six males and five females, with a median age of 43 years (range;14–53), with nine in soft tissue and two in bone. Tumor size ranged from 3.5 cm to 20.0 cm (mean: 9.8 cm). Most cases showed sheets of undifferentiated round- to oval-shaped cells. Two cases showed an Ewing-like pattern, and one case showed spindle cells in a fibrotic stroma transitioning to epithelioid cells. Necrosis was present in nine cases, and mitotic count ranged from 2 to 38/ 10HPFs (mean = 14.2). CD99 was positive in (10/11) cases and WT-1 in (6/9). NKX2.2, S100, and MDM2 were positive in rare cases. *CIC::DUX4* fusion was detected in four cases. FISH for *CIC* gene rearrangement was positive in seven cases, two of them confirmed by methylation analysis. Metastasis at diagnosis was common (n = 8), primarily in the lungs, with later metastasis to the brain and bone. At time of final analysis, eight patients died within a median of 10 months (range: 1–19 months), while three were alive, two with stable disease (for a period of 6 and 25 months) and one with progression after 10 months. Significant correlation was seen between overall survival and the presence of metastasis at diagnosis (*p* value = 0.03). **Conclusions**: *CIC*-rearranged sarcomas are rare, high-grade tumors with predilection for soft tissue. Misdiagnosis is frequent, necessitating molecular confirmation. These tumors are treatment-resistant, often present with lung metastasis, and carry a poor prognosis, especially with initial metastasis.

## 1. Introduction

In the latest WHO classification, undifferentiated small round cell sarcomas of bone and soft tissue are divided into four distinct categories: Ewing sarcoma (ES), round cell sarcomas with *EWSR*1::non-ETS fusions, sarcomas with *BCOR* genetic alterations, and *CIC*-rearranged sarcomas [1]. *CIC*-rearranged sarcomas are highly aggressive malignancies that predominantly arise in the soft tissues of children and young adults [2]. While they share some morphological similarities with Ewing sarcoma, molecular studies indicate that *CIC*-rearranged sarcomas have distinct pathogenesis. Histologically, *CIC*-rearranged sarcomas are characterized by proliferation of undifferentiated round to oval tumor cells with variable myxoid background. The differential diagnosis includes Ewing sarcoma, BCOR-altered sarcoma, sarcoma with EWSR-1::non-ETS fusion, and other round cell tumors. The diagnosis of *CIC*-rearranged sarcoma requires combining histological features with immunohistochemical stains that function as surrogate markers for the gene fusion. The gold standard for diagnosis is detecting the *CIC* gene rearrangement by molecular tests. By definition, these tumors have rearrangement of the *CIC* gene with variable gene fusion partners. The *CIC::DUX4* fusion is the most common (seen in 95% of cases) and is associated with chromosomal translocations t(4;19)(q35;q13) or t(10;19)(q26;q13) [1,3,4,5,6,7]. Other rare examples include non-DUX4 partner genes such as *NUMT1*, *NUTM2A*, *FOXO4*, *LEUTX*, and *CREBBP* [8,9,10,11]. CIC oncogenic fusions upregulate its targets—including the ETS variant transcription factor (ETV) 1/4/5 PEA3 family of ETS transcription factors, CCND2, and MUC5AC [3,12,13]—promoting tumor development and progression. The treatment of most patients with *CIC*-rearranged sarcomas involves multimodal approaches, including a combination of chemotherapy, surgery, and/or radiation, typically modeled on protocols established for Ewing sarcoma or soft tissue sarcomas. However, there is currently no standardized treatment regimen or prospective randomized controlled trials specific to *CIC*-rearranged sarcomas, nor there is a consensus on how to stratify these patients for optimal therapy [14,15,16]. To the best of our knowledge, there are no reported case series of *CIC*-rearranged sarcoma from the Middle East. This study presents a series of eleven patients diagnosed with *CIC*-rearranged sarcoma, highlighting their clinicopathological features, treatment approaches, and outcomes.

## 2. Materials and Methods

A retrospective review was conducted on patients diagnosed with *CIC*-rearranged sarcoma and treated at the King Hussein Cancer Center (KHCC) between May 2019 and December 2024. Patients were identified via an electronic medical record search using the term “*CIC*-sarcoma.” Eligible cases had a confirmed histopathological diagnosis of *CIC*-rearranged sarcoma, validated through pathology review and ancillary molecular testing. All patients were managed and followed at KHCC under the guidance of a multidisciplinary sarcoma team.

Clinical data, including disease presentation, tumor progression, metastatic status at diagnosis and follow-up, imaging findings, treatment modalities, and overall survival (OS), were retrospectively extracted from medical records. The histopathologic and molecular characteristics were reviewed. Descriptive statistics including counts and percentages for categorical variables and means and medians for continuous variables were used. OS was defined as the interval from pathological diagnosis to either death or the most recent follow-up. Kaplan–Meier survival analysis was performed using GraphPad Prism (Version 10.4.1 for macOS, GraphPad Software, Boston, MA, USA, https://www.graphpad.com/, accessed on 24 January 2025). The correlation between tumor size and metastatic status at diagnosis with OS was analyzed using the Mantel–Cox log-rank test. The KHCC Institutional Review Board (IRB) granted approval for the retrospective extraction and analysis of clinical data for this study.

## 3. Results

The clinical and pathological characteristics of the eleven identified cases are summarized in Table 1 and Table 2. The cohort included six males (55%) and five females (45%), yielding a male-to-female ratio of 1.2:1. The age at diagnosis ranged from 14 to 53 years, with a mean of 37.4 years and a median of 43 years. Tumor localization included soft tissue (n = 9) and bone (n = 2). The soft tissue tumors were located in the mesentery (n = 1), paraspinal region (n = 1), gluteus maximus muscle (n = 1), right frontal brain lobe (n = 1), groin (n = 1), upper extremity/axilla (n = 1), and thigh (n = 3), while the bone tumors were found in the clavicle (n = 1) and iliac bone (n = 1).

Seven cases were initially diagnosed at referring facilities with the following diagnoses: malignant high-grade sarcoma with myxoid and round cell areas (n = 1), extraskeletal Ewing sarcoma (n = 2), low-to-intermediate-grade sarcoma (n = 1), sarcoma not otherwise specified (n = 2), and synovial sarcoma (n = 1). One case (Case 2) was initially diagnosed as a round cell liposarcoma at KHCC but later reclassified as *CIC::DUX4* sarcoma following RNA sequencing.

The presenting symptoms were pain (n = 6), mass (n = 8), and status epilepticus (n = 1). Tumor sizes ranged from 3.5 to 20.0 cm (mean: 9.8 cm), with only two cases measuring <5.0 cm. Metastatic disease at diagnosis was observed in eight cases, primarily involving the lungs (n = 8), including one with concurrent bone metastasis. Subsequent metastatic spread included brain involvement in three cases and lung involvement in one additional case. At the time of analysis, two patients remained metastasis-free.

**Figure 1:**
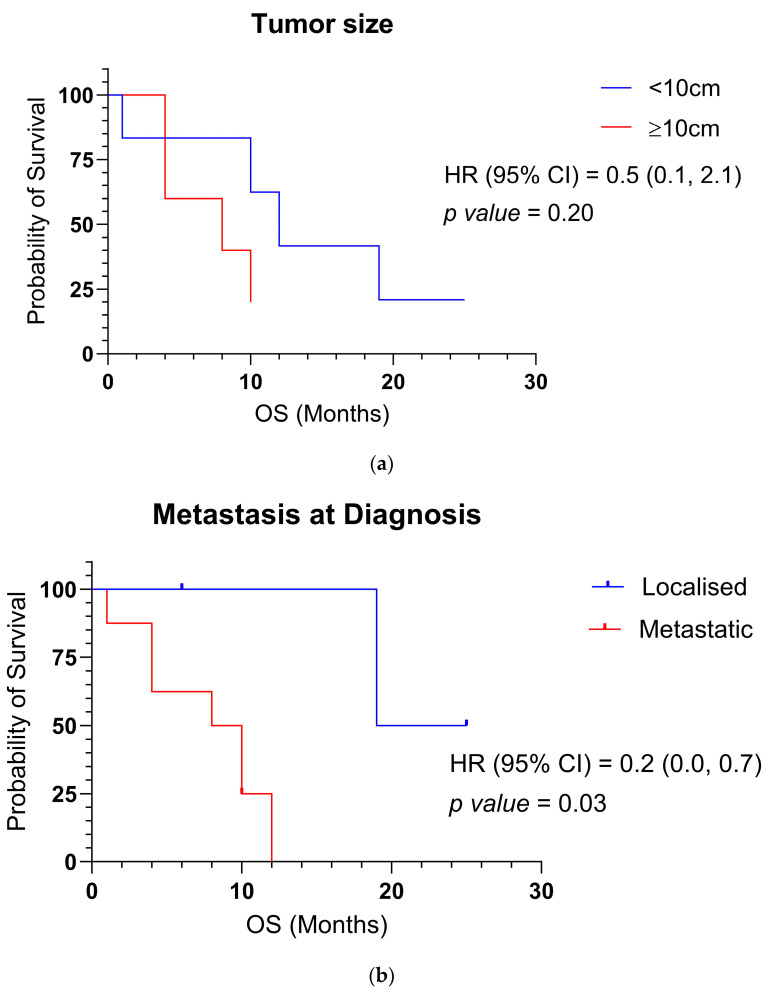
(**a**) Overall survival and its correlation with tumor size; (**b**) overall survival and its correlation with the presence of metastasis at diagnosis.

Treatment strategies were either palliative or curative, employing chemotherapy (predominantly Ewing sarcoma-based regimens), radiotherapy, and/or surgical excision following the recommendations of our sarcoma multidisciplinary team. Eight patients died from disease progression within 1–19 months of diagnosis (median: 9 months; mean: 8.5 months). One patient experienced disease progression at 10 months and was referred to palliative care. Two patients remained alive: one (Case 11) was continuing active chemotherapy, while the other (Case 7) had undergone surgical excision and received a single cycle of chemotherapy, which was discontinued due to toxicity. The median survival time for localized and metastatic disease was 19 and 10 months, respectively. Statistical analysis revealed no significant correlation between OS and tumor size (≥10 cm, *p* value = 0.20. However, the presence of metastasis at time of diagnosis was significantly associated with poorer OS (*p* = 0.03). Overall survival and its correlation with tumor size and the presence of metastasis at time of diagnosis are summarized in Figure 1a,b.

**Figure 2:**
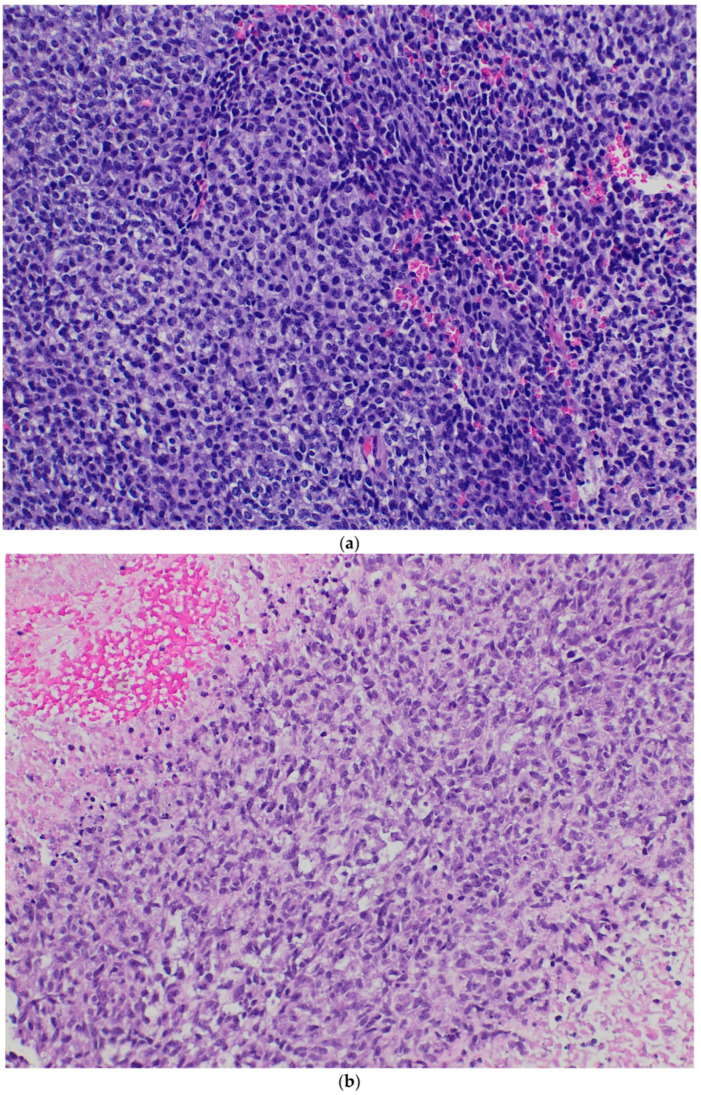

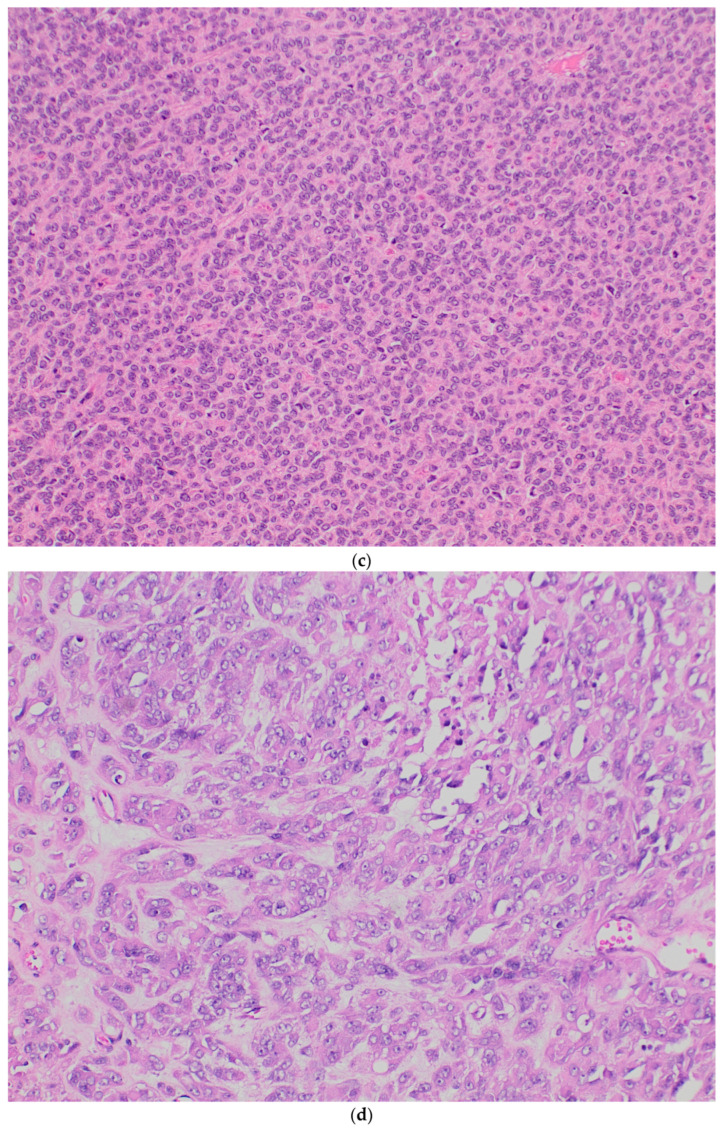

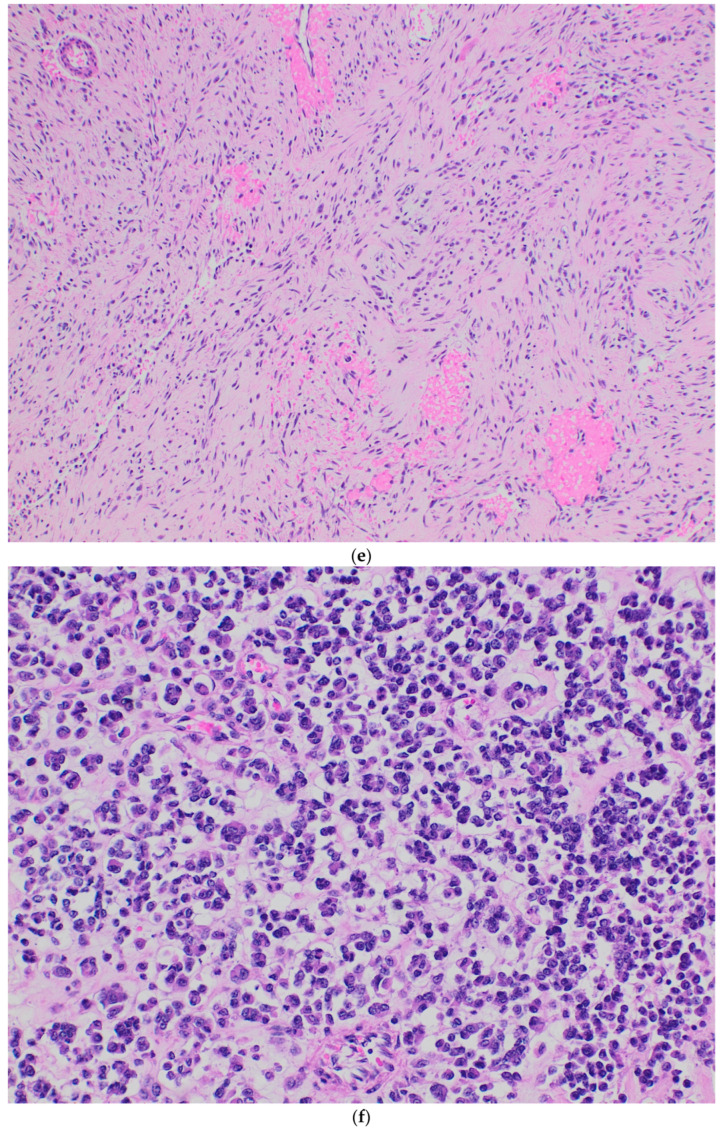
*CIC*-rearranged sarcoma. (**a**) Diffuse, solid sheets of small undifferentiated malignant tumor cells. Hematoxylin and eosin, 200×. (**b**) Focal spindled cell morphology. Hematoxylin and eosin, 200×. (**c**) Ewing-like morphology. Hematoxylin and eosin, 200×. (**d**) Epithelioid to rhabdoid tumor cells with higher degree of nuclear pleomorphism. Hematoxylin and eosin, 200×. (**e**,**f**) Biphasic pattern with areas showing short-spindle cells in a fibrotic background (**e**)—hematoxylin and eosin, 100×—to a more cellular area with tumor cells showing epithelioid morphology with higher nuclear grade (**f**)—hematoxylin and eosin, 200×.

The histological features of the eleven cases are summarized in Table 2. The tumors predominantly exhibited solid sheet-like arrangements of undifferentiated, round- to oval-shaped cells (Figure 2a). Other less commonly observed tumor arrangements included short-spindle cell fascicles (Figure 2b), nests, cords, trabeculae, and sieve-like areas with focal microcyst formation. Myxoid background was observed in three cases. The chromatin pattern ranged from dark with inconspicuous nucleoli to vesicular with prominent nucleoli. The mitotic count ranged from 2 to 38/10HPFs (mean: 14.2), excluding two cases with limited assessable material. Necrosis was present in nine cases. Nuclear pleomorphism was typically mild in most cases but more pronounced than in Ewing sarcoma. Two cases exhibited pure Ewing-like morphology manifested by sheets of monotonous round tumor cells (Cases 7 and 11) (Figure 2c), while two others demonstrated significant pleomorphism with epithelioid-to-rhabdoid cells (Figure 2d), one following neoadjuvant therapy. This particular case exhibited a biphasic pattern showing transition from short-spindle cells in a fibrotic background (Figure 2e) to more cellular areas with tumor cells exhibiting epithelioid morphology, higher nuclear grade, and significant pleomorphism (Figure 2f).

## 4. Discussion

*CIC*-rearranged sarcoma is a rare and aggressive subtype of undifferentiated small round cell sarcoma, recently recognized as a distinct entity in the latest WHO classification of soft tissue and bone tumors. Characterized by its aggressive clinical behavior and resistance to conventional treatment modalities, this neoplasm presents significant diagnostic and therapeutic challenges. To date, no prospective clinical studies have established optimal treatment strategies, and the prognosis remains poor. Existing knowledge is primarily derived from case series, clinicopathological studies, and case reports. Herein, we present our institutional experience in diagnosing and managing this tumor, reporting the pathological features and clinical outcomes of eleven cases treated between 2019 and 2024. Our report is concordant with the international literature, with poor outcome of cases, as most patients presented with metastasis and experienced disease progression leading to death despite treatment. Additionally, many of these cases were misdiagnosed initially, and were correctly diagnosed after careful pathological review and confirmed—at time of diagnosis or later—using advanced molecular testing including FISH, RNA sequencing, and DNA methylation-based tumor classification.

Histologically, *CIC* sarcoma shows variable histological patterns including diffuse sheets of undifferentiated cells, reticular or sieve-like arrangements with variable myxoid background, focal fascicular proliferation of spindle cells, pure round cell morphology indistinguishable from Ewing sarcoma, intersecting storiform pattern, and some rare variants including purely myxoid and purely epithelioid tumors [1,2,17]. Most of our cases showed sheets of undifferentiated round to oval shaped cells with occasional short-spindle cell areas. In rare instances, epithelioid to rhabdoid cells were observed. One case exhibited a biphasic pattern, transitioning from short-spindle cells in a fibrotic stroma to a higher-grade epithelioid component with cellular pleomorphism. This pattern was identified in a resected specimen post-neoadjuvant chemotherapy. Although the fibrotic stroma could theoretically represent post treatment changes, the absence of necrosis, dropout areas, or inflammatory infiltrates argues against this interpretation. We propose that this represents a rare histological pattern of *CIC*-rearranged sarcoma that, to the best of our knowledge, was not previously described in the literature.

A pure Ewing-like morphology was infrequently observed, which may pose a diagnostic challenge, particularly in tumors demonstrating diffuse CD99 and NKX2.2 positivity, as in Case 7. Although rare, NKX2.2 positivity was reported by Hung et al. [18], which may lead to inappropriate diagnosis of Ewing sarcoma. However, our case showed positive *CIC* and negative *EWSR-1* gene rearrangements by FISH, confirming the diagnosis of *CIC*-rearranged sarcoma.

The IHC profile of our cases aligned with previously reported staining patterns of *CIC*-rearranged sarcomas. CD99 and WT-1 were the most commonly expressed markers, with CD99 exhibiting focal staining in most cases. Less commonly observed patterns of CD99 expression included diffuse membranous and cytoplasmic para-nuclear dot-like staining. WT-1 was positive in six of nine tested cases. Additional markers, including S100, NKX2.2, MDM2 (the latter seen in one case and in another recently diagnosed case not included in this series), and ERG were rarely expressed. For diagnostic purposes, we confirm that focal expression of CD99 in a small round cell sarcoma should raise the differential diagnosis of *CIC*-rearranged sarcoma. WT-1 immunostain is a sensitive marker for this tumor, and when it is positive, it strongly suggests the diagnosis which may be confirmed using molecular tests.

*CIC* gene rearrangements can be detected using various molecular techniques. Among them, CIC break-apart FISH is widely employed because it does not require prior knowledge of the fusion partner. However, this method has limited sensitivity, with approximately 14–25% of cases yielding false-negative results despite the presence of a *CIC* gene fusion [19,20]. This limitation is particularly evident in tumors harboring *CIC::DUX4* fusions [17]. The exact cause of these false negative results is unclear but is likely related to complex or cryptic chromosomal rearrangements [21]. Despite its limitations, FISH is generally inexpensive, has a short turnaround time, and can be performed on small tissue samples.

Targeted RNA sequencing has emerged as a more sensitive diagnostic tool for *CIC*-rearranged sarcoma. It not only detects the specific gene fusion but can also identify novel gene fusion partners and provide gene expression information [21]. Nevertheless, false negative result can still occur, especially with *CIC::DUX4* fusions, due to limitations in fusion-calling algorithms. These errors often arise when chimeric reads are incorrectly filtered out as noise, owing to the repetitive nature of the *DUX4* sequence [17,21]. In such cases, manual review of RNA sequencing data or analysis of the expression profiles of *ETV1*, *ETV4*, and *ETV5* may be diagnostically helpful.

*CIC*-rearranged sarcoma can also be confirmed using DNA methylation-based tumor classification. However, this approach still requires systematic validation and is currently limited to large academic centers with access to specialized bioinformatics resources and expertise. It is important to note that tumors with atypical fusions, such as *CIC::LEUTX*, may not cluster closely with other *CIC*-rearranged tumors. Additionally, tumors involving *ATXN1* rearrangements—such as *ATXN1::NUTM1* and *ATXN1::NUTM2*—may cluster with *CIC*-rearranged sarcomas by methylation profiling, despite lacking a *CIC* gene fusion [21]. These rare tumors, which have been primarily described in the central nervous system, share overlapping phenotypic or methylation features with *CIC*-rearranged sarcomas [17]. Methylation profiling may be particularly valuable in cases where FISH and RNA sequencing results are negative, especially when the index of suspicion is high for *CIC*-rearranged sarcoma by morphology and IHC.

For our cases, molecular confirmation of *CIC* gene rearrangement was achieved using FISH, RNA sequencing, or DNA methylation-based tumor classification. While molecular testing remains the gold standard for diagnosis, we propose that in resource-limited settings, the combination of clinical findings, histological features and IHC markers may suffice for accurate diagnosis. In fact, some of our cases were confirmed retrospectively following the introduction of those tests into our practice. Recently reported IHC markers such as ETV4, DUX4, and MUC5AC further aid in the diagnosis, along with other markers such as WT1, CD99, and ERG. The sensitivity and specificity of WT1 are 95% and 81%, respectively, whereas diffuse ETV4 expression has a sensitivity and specificity of 90% and 95%, respectively. ETV4 expression, along with at least focal WT1 expression is helpful in distinguishing *CIC*-rearranged sarcoma from its differential diagnosis [22,23], although some challenges maybe encountered in optimizing the ETV4 antibody. Recently, DUX4 and MUC5AC were introduced as helpful diagnostic stains [24,25]; the latter is reported to be typically expressed in scattered tumor cells and—despite its low specificity—may be particularly useful given its wide availability.

Prognostic factors influencing OS may include tumor size, treatment modality, and metastatic status at diagnosis. Murphy et al. reported a survival advantage in three out of nine patients who had tumors smaller than 5.0 cm and presented with localized disease, although some of those patients also had complete surgical resection with negative margins and received adjuvant radiation therapy [26]. Connolly et al. described a median overall survival of 40.6 months for localized disease versus 12.6 months for metastatic cases [11]. The French sarcoma group reported a large retrospective series of 79 cases of *CIC*-rearranged sarcoma comparing two patient groups: one treated as classical Ewing sarcoma and the other as high-grade soft tissue sarcoma [15]. They found no significant difference in median overall survival between the two groups but showed that all patients who presented with metastasis at diagnosis and were treated as high-grade soft tissue sarcoma died from the disease compared to some patients from the Ewing sarcoma group who remained alive and in complete remission [15]. Other reports have also confirmed the potential benefit of the Ewing sarcoma therapy protocol in *CIC*-rearranged sarcoma [27,28], including in the metastatic setting [11,29]. However, some reports questioned the benefit of neoadjuvant chemotherapy, particularly given the considerable risk of disease progression or metastasis due to the tumor’s aggressive nature. This was signaled by Italiano et al. and Connolly et al., who reported disease progression during neoadjuvant chemotherapy [5,11]. In addition, Antunescu et al. reported inferior survival for patients treated with neoadjuvant therapy compared with surgical excision, although patients in that group had larger tumors [2]. Although some cases in our cohort suggest a possible benefit of upfront surgery over neoadjuvant chemotherapy in selected situations, this observation is hypothesis-generating and should be evaluated alongside other factors, ideally through discussion within a multidisciplinary sarcoma team. Given the small sample size and retrospective nature of our study, no definitive conclusions can be drawn. Further research is warranted to assess this potential treatment approach.

Our findings underscore the aggressive clinical behavior of *CIC*-rearranged sarcoma, as evidenced by the fact that eight patients presented with metastatic disease at diagnosis, most commonly to the lungs. Notably, four patients developed brain metastases—a rare occurrence in sarcomas—highlighting a possible tumor’s predilection for atypical metastatic sites. These results support prior observations that the presence of metastasis at initial presentation is associated with significantly worse outcome. Although limited by the sample size, our data demonstrate a survival disadvantage in patients with metastases at diagnosis compared to those with localized disease. Conversely, two patients with localized disease are currently disease-controlled and merit further discussion, as they may offer insights into potential prognostic or therapeutic factors influencing favorable outcomes. Case 7 presented with a 7 cm mesenteric mass that was resected with negative margins. Despite receiving only one cycle of adjuvant chemotherapy, she remains disease-free. This case is peculiar in several aspects: it shares histological and immunohistochemical features with Ewing sarcoma, has a low mitotic count with no necrosis—suggestive of a lower-grade neoplasm—and remains disease-free following only surgical resection, which is atypical for both Ewing sarcoma and *CIC*-rearranged sarcoma. We suggest that this tumor demonstrates favorable biological behavior probably because of its relatively “low grade” histologic features and the complete surgical resection. This might also support the previously mentioned observation of better outcomes when surgical excision is performed prior to neoadjuvant chemotherapy. The second case, Case 11, is for a 14-year-old male with a history of treated pre-B-cell acute lymphoblastic leukemia who presented with a 3.5 cm frontal lobe brain mass. Following near gross total resection, he received adjuvant ICE chemotherapy with controlled disease. Similar to the previous case, surgical excision prior to the administration of neoadjuvant chemotherapy may suggest a potential benefit of upfront surgical resection. However, longer follow up is needed for this patient to truly assess the course of his disease.

Some limitations in this study should be acknowledged, including the small sample size, retrospective design, and single-institution setting, which may introduce selection bias and restrict broader applicability. Due to the limited sample size, our study may lack sufficient statistical power to draw definitive conclusions regarding the most appropriate treatment modality and the presence of significant correlations between clinical or pathological features with overall survival. Although the *p*-values were calculated to assess statistical significance between some clinical and pathological factors and OS, the small cohort size (n = 11) limits the robustness of these results and reduces confidence in the generalizability of our findings. To make definitive conclusions, our observations should be confirmed in studies with larger sample sizes. Additionally, the predominance of metastatic cases constrained a comprehensive analysis of therapeutic responses. Nevertheless, this is the first reported case series from the Middle East, offering needed insights into the diagnosis and management of this rare tumor in a resource-limited setting. We identified a potentially novel histologic pattern and highlighted the importance of integrating pathologic features, cost-effective ancillary studies, and clinical correlations for accurate diagnosis. Furthermore, we presented some cases that were associated with better clinical course and discussed the potential clinical and pathological findings that might have contributed to the favorable outcome. We also highlighted the possible negative impact on prognosis when patients present initially with metastasis. In conclusion, *CIC*-rearranged sarcoma is a highly aggressive neoplasm, requiring specialized diagnostic process and advanced care. However, prognosis remains poor, which necessitates establishing clinical trials and prospective studies to enhance treatment options and outcome.

## Figures and Tables

**Table 1:** Clinical features of eleven patients with *CIC*-rearranged sarcomas.

Case	Age (Years)/Gender/Symptoms and Clinical Features	Site	Size (cm)	Radiological Features	Metastasis	Treatment	Outcome
1	52/F/mass	Left thigh	8.3	MRI: Large subcutaneous left thigh soft tissue mass suggestive of soft tissue sarcoma.	Lung at presentation, then brain metastasis	Ifosfamide, cisplatin, and etoposide at another institution. Palliative radiotherapy.	DOD 10 months
2	42/F/mass	Right axilla/infra clavicular	7.0	CT scan: Large well-defined soft tissue density mass lesion in the right upper axilla anterior to the shoulder joint, scapula, and upper ribs.	No metastasis at presentation, then lung metastasis	R2 excision, adjuvant radiotherapy. Chemotherapy (VDC-IE).	DOD 19 months
3	25/M/pain and mass	Left thigh	15.0	MRI: Osseous mass at left iliac bone and acetabulum with post-gadolinium enhancement, associated with a soft tissue component at both sides of the iliac wing.	Lung at presentation, then brain metastasis	Chemotherapy (VDC-IE), radiotherapy.	DOD 8 months
4	53/M/pain and mass	Left groin	7.4	CT scan: Infiltrative left iliopsoas muscle mass.	Lung at presentation, then brain metastasis	Chemotherapy (VDC-IE) followed by surgical excision and adjuvant radiotherapy.	DOD 12 months
5	47/F/mass	Left thigh	10.2	MRI: A heterogeneous enhancing soft tissue mass with hemorrhagic contents at the medial anterior compartment of the left thigh encasing the superficial femoral artery.	Lung metastasis at presentation	Chemotherapy (VDC-IE), neoadjuvant radiotherapy, excision with negative margins.	DOD 10 months
6	47/M/mass	Dorsum of the right foot	4.8	MRI: A soft tissue mass in the second web space with dorsal and plantar components.	Lung and bone metastasis at presentation	Chemotherapy (VDC), radiotherapy for spine metastasis.	DOD 1 month
7	43/F/abdominal pain	Mesentery	7.0	CT scan: Left pelvic mesenteric mass abutting the left ovary, sigmoid, and anterior abdominal wall without definite invasion.	No	Excision (margins negative), one cycle VDC.	NED 25 months
8	19/M/mass with restriction of shoulder movement	Right shoulder	20.0	MRI: An aggressive mass in the shoulder with destruction of the distal clavicle and acromion with a large extra osseous soft tissue component occupying most of the ventral and superolateral aspect of the shoulder. It showed heterogeneous enhancement with a large necrotic component.	Lung metastasis at presentation	VDC-IE.	DOD 4 months
9	53/F/back pain	Left paraspina-l at the level of L2-L4 levels	10.0	MRI: A relatively large heterogeneous left paraspinal soft tissue mass invading the posterior elements of the lower lumbar vertebrae and showing intraspinal extension compressing the thecal sac with focal canal stenosis.	Lung metastasis at presentation	Chemotherapy (VDC-IE), L3-L4 spine decompression, radiotherapy.	AWD 10 months
10	16/M/pain, rapidly progressing mass	Left gluteus maximus muscle	15.0	MRI: Large multilobulated mass within the left gluteus maximus, with heterogeneous enhancement and hemorrhagic components.	Lung metastasis at presentation	Chemotherapy (VDC-IE).	DOD 4 months
11	14/M/kno-wn case of pre-B-cell ALL t (12;21) post-BMT presented with status epilepticus. On exam, the patient had left-sided upper limb weakness and diffuse skin nevi but no café au lait spots	Brain/right frontal lobe	3.5	MRI: Right frontal cortical and subcortical complex mass (cystic and solid).	No	Surgery, chemotherapy (ICE).	AWD 6 months

ALL: acute lymphoblastic leukemia; BMT: bone marrow transplant; F: female; VDC: vincristine, doxorubicin, cyclophosphamide; IE: ifosfamide, etoposide; ICE: ifosfamide, carboplatin, etoposide; DOD: died of disease; AWD: alive with disease; NED: no evidence of disease.

**Table 2:** Pathological and molecular features of 11 cases of *CIC*-rearranged sarcoma.

Case	Referred/Outside Diagnosis	Histologic Pattern	IHC Profile	Molecular Findings
1	Sarcoma, not otherwise specified	Sheets of small, undifferentiated round cells with dark nuclei.	Positive: FLI-1, BCL2, NSE, and SMA. Focally positive: CD99 and WT1. Negative: S100, desmin, CK-MNF, and myogenin.	FISH for *CIC* fusion is positive.
2	High-grade sarcoma with myxoid and round cell areas.	Nests, cords, and trabeculae of undifferentiated round- to oval-shaped cells with vesicular nuclei and prominent nucleoli in a fibrotic and myxoid background. Occasional cells with clear cytoplasm and a few cells with epithelioid and rhabdoid features.	Positive: FLI-1 and CD56. Focally positive: CD99 and synaptophysin. Negative: CD99, CK-MNF, desmin, S100, SOX10, TLE-1, and ERG.	*CIC::DUX4* by RNA sequencing.
3	Not applicable	Sheets of small, undifferentiated round to oval-shaped and short-spindled cells with dark nuclei.	Positive: FLI-1 and WT-1. Focally positive: CD99. Negative: NKX2.2, panCK, desmin, synaptophysin, S100, SATB2 and ERG.	*CIC::DUX4* by RNA sequencing.
4	Low- to intermediate-grade sarcoma	Sheets of small, undifferentiated round- to oval-shaped cells with dark nuclei. Post neoadjuvant: Biphasic pattern with spindle cell areas showing abrupt transition to atypical epithelioid cells.	Diffusely positive: CD99 and WT-1. Dot-like positivity: EMA and CK-MNF. Negative: S100, desmin, synaptophysin, NKX2.2, and STAT6.	*CIC::DUX4* by RNA sequencing.
5	Extraskeletal Ewing sarcoma	Sheets of small, undifferentiated round cells with dark nuclei.	Diffusely positive: CD99, WT1, and ERG. Negative: NKX2.2, synaptophysin, and BCOR.	*CIC::DUX4* by RNA sequencing.
6	Synovial sarcoma	Sheets of small, undifferentiated round- to oval-shaped and short-spindled cells with focal myxoid background.	Focally positive: CD99 and SATB2 Negative: ERG, BCOR, NKX2.2, synaptophysin, WT1, S100, panCK, SS18, desmin, STAT6, and CK-MNF. Retained nuclear expression: H3K27me3	FISH for *CIC* rearrangement is positive. Methylation confirmed the diagnosis.
7	Extraskeletal Ewing sarcoma	Sheets of monotonous small, undifferentiated round cells, very similar to Ewing sarcoma.	Diffusely positive: CD99, NKX2.2, and S100. Negative: DOG-1, synaptophysin, SS18, WT-1, panCK, CK-MNF, desmin, and SALL4. Retained nuclear expression: INI1.	FISH for *CIC* rearrangement positive. FISH for EWSR-1 gene rearrangement is negative.
8	Not applicable	Sheets of oval to short-spindled cells with dark nuclei.	Focally positive: CD99, WT1, and ERG. Negative: PanCK, Desmin, S100, NKX2.2, BCOR, SS18, and SATB2.	FISH for *CIC* rearrangement positive.
9	Not applicable	Sheets, short-spindled and anastomosing cords of round- to oval-shaped cells with vesicular nuclei and prominent nucleoli. Microcyst formation and focal myxoid background.	Positive: MDM2. Dot-like positivity: CD99. Focally positive: EMA. Negative: PanCK, CK-MNF, WT-1, CD31, S100, BCOR, S100, and STAT-6.	FISH for *CIC* rearrangement positive.
10	Sarcoma, not otherwise specified	Sheets of small, undifferentiated round cells with dark nuclei.	Focally positive: CD99 and WT1. Negative: SATB2, MyoD1, and SOX10.	FISH for *CIC* rearrangement positive.
11	Not applicable	Sheets of round to oval cells, similar to Ewing sarcoma.	Diffusely positive: CD99 and FLI-1. Focally positive: Synaptophysin. Negative: NKX2.2, BCOR, Desmin, STAT-6, SATB2, SOX10, S100, and panCK. Retained nuclear expression: INI1 and BRG1.	FISH for *CIC* rearrangement positive. Methylation confirmed the diagnosis.

## Data Availability

Data is contained within the article.
